# Optimization of Zein-Casein-Hyaluronic Acid Nanoparticles Obtained by Nanoprecipitation Using Design of Experiments (DoE)

**DOI:** 10.1021/acsomega.4c11636

**Published:** 2025-03-28

**Authors:** Tatiane Patrícia Babinski, Ariane Krause Padilha Lorenzett, Jeferson Ziebarth, Vanderlei Aparecido de Lima, Rubiana Mara Mainardes

**Affiliations:** † Laboratory of Nanostructured Formulations, Universidade Estadual do Centro-Oeste, Élio Antonio Dalla Vecchia St, 838, 85040-167 Guarapuava, PR, Brazil; ‡ Chemistry Department, Universidade Tecnológica Federal do Paraná, 85503-390 Pato Branco, PR, Brazil; § Department of Pharmacy, Universidade Estadual do Centro-Oeste, Élio Antonio Dalla Vecchia St, 838, 85040-167 Guarapuava, PR, Brazil

## Abstract

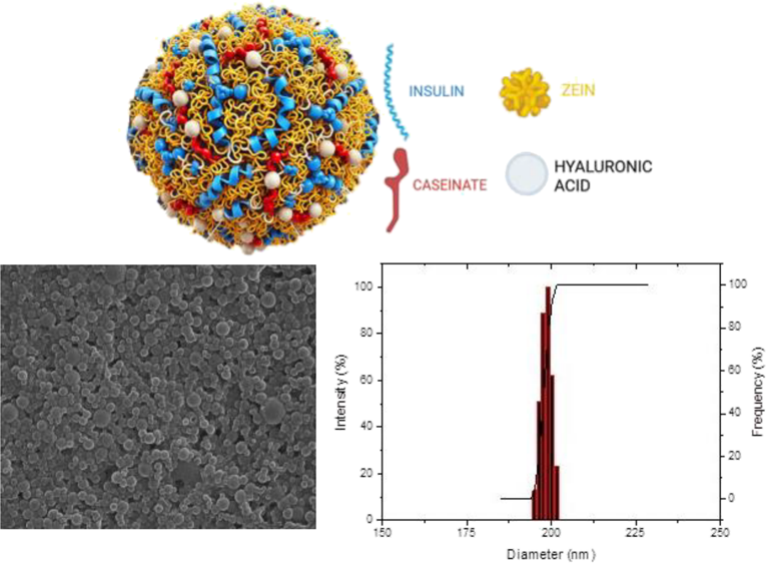

Zein-based nanoparticles offer significant potential as carriers for drug delivery due to their biocompatibility. However, optimizing their formulation is essential to achieving efficient encapsulation and stability. This study aimed to optimize the formulation of zein-casein-hyaluronic acid-based nanoparticles for the encapsulation of a hydrophilic drug, focusing on achieving favorable physicochemical properties for oral drug delivery applications. A factorial experimental design was employed to evaluate the influence of key formulation parameters, including zein concentration, hyaluronic acid concentration, sodium caseinate concentration, and the organic-to-aqueous phase (O/W) ratio. Particle size (PS), polydispersity index (PDI), zeta potential, and encapsulation efficiency (EE) were analyzed as response variables. Multivariate analyses, such as hierarchical cluster analysis and principal component analysis, were performed to explore the relationships between formulation parameters and nanoparticle properties. Model validity was confirmed by using ANOVA and residual analysis. Optimized nanoparticles exhibited a PS of 217 ± 5 nm, PDI of 0.077 ± 0.022, zeta potential of −24.7 ± 1.9 mV, and EE of 31% ± 4. The nanoparticles displayed a monomodal size distribution and a spherical morphology. Multivariate analyses revealed that the O/W ratio and zein concentration were the most influential factors, while sodium caseinate played a crucial stabilizing role. The desirability function yielded a high score (*D* = 0.9338), confirming the robustness of the optimization process. Stability studies demonstrated that refrigeration at 8 °C preserved the nanoparticles’ physicochemical properties over 180 days. This study underscores the power of experimental design as a tool to refine nanoparticle formulations, paving the way for more efficient drug delivery systems and unlocking new possibilities for the oral administration of hydrophilic compounds.

## 1 Introduction

Nanoparticles have been widely investigated in materials science due to their unique properties, such as a high surface-area-to-volume ratio, tunable size, and the ability to encapsulate and release bioactive compounds in a controlled manner. These attributes make them ideal candidates for diverse applications, including drug delivery, nutrient encapsulation, and the design of functional foods, offering significant advantages in oral administration and alternative routes.^1−3^ The adaptability of nanoparticles lies in their capacity for structural modifications, which can enhance the solubility, bioavailability, and stability of the encapsulated compounds. Such versatility has positioned nanoparticles as valuable tools in the pharmaceutical, cosmetic, and food industries.^4,5^

Among the diverse materials used for nanoparticle synthesis, zein, a hydrophobic prolamin protein derived from corn, has emerged as a promising candidate due to its favorable characteristics. Zein is biocompatible, biodegradable, and classified as a Generally Recognized as Safe (GRAS) substance by regulatory agencies, making it particularly suitable for applications in food and pharmaceutical products.^6,7^ One of its most notable properties is its ability to self-assemble in aqueous environments, forming nanoparticles through antisolvent precipitation. This process involves the reduction of ethanol concentration, inducing the precipitation of zein and facilitating nanoparticle formation.^8,9^

Despite these advantages, the inherent hydrophobic nature of zein presents challenges, particularly in maintaining colloidal stability in aqueous environments. This limitation often results in nanoparticle aggregation, reducing the efficiency and functionality of the delivery system.^10−14^ Addressing this challenge requires innovative approaches, such as the incorporation of stabilizers like sodium caseinate, hyaluronic acid (HA), gum arabic, sodium alginate, chitosan, and poly(vinyl alcohol), which enhance particle dispersion and stability.^4,15,16^ Additionally, the optimization of formulation parameters, including zein concentration, organic-to-aqueous phase ratio, and the choice of stabilizers, plays a crucial role in achieving nanoparticles with desirable physicochemical properties such as size uniformity, encapsulation efficiency (EE), and sustained release profiles.^17,18^

The complexity of these interactions underscores the importance of employing systematic optimization methodologies. Factorial design, a core aspect of the Design of Experiments (DoE), offers a robust framework for the simultaneous evaluation of multiple factors and their interactions. This statistical approach not only facilitates the identification of optimal formulation conditions but also provides critical insights into how individual variables influence nanoparticle characteristics, such as stability, EE, and drug release kinetics.^19,20^

In this study, we aim to develop and optimize zein-casein-HA-based nanoparticles for the encapsulation of a model hydrophilic drug using factorial experimental design. By leveraging DoE methodologies, we seek to provide a comprehensive understanding of the key formulation parameters, advancing the potential of zein nanoparticles in pharmaceutical and nutraceutical applications.

## 2 Materials and Methods

### 2.1 Materials

Zein, sodium caseinate, formic acid (HPLC grade), and acetonitrile (HPLC grade) were purchased from Sigma-Aldrich (St. Louis, MO, USA). HA (8–15 kDa) was obtained from Contipro (Doln *D*obrou, Pardubice Region, Czech Republic). Insulin (INS) (100 UI/mL) was purchased from Novolin R (Novo Nordisk, São Paulo, Brazil). Absolute ethanol was purchased from Synth (São Paulo, SP, Brazil).

### 2.2 Optimization of Zein Nanoparticle Synthesis Using Experimental Design (DoE)

A complete factorial design with central points was employed to evaluate the influence of independent variables on dependent variables during the production process. This approach aimed to optimize the formulation parameters for an ideal nanoparticle system. Key parameters affecting nanoparticle production were selected based on prior literature.^11,21,22^ The experimental matrix was designed and randomized using Minitab version 18, and statistical analyses (ANOVA and multilinear regression) were performed using Statistica to assess the model significance and interaction effects.

Four independent variables were evaluated: Zein concentration (X1), HA concentration (X2), sodium caseinate concentration (X3), and organic-to-aqueous phase ratio (O/W ratio) (X4). The dependent variables included mean diameter (R1), polydispersity index (PDI) (R2), zeta potential (ZP) (R3), and drug encapsulation efficiency (%EE) (R4). Twenty-two experiments, including six central points, were conducted to estimate pure error and interaction terms. The levels of all factors and the variables are presented in Table 1. The coded factors and levels are provided in the Supporting Information. The goal was to obtain nanoparticles with a particle size (PS) below 300 nm, PDI below 0.2, ZP of approximately −30 mV, and an EE of at least 30%.

**Table 1:** Factors and Levels Used in the Factorial Design Experiment to Obtain Zein Nanoparticles

		levels		
factor	independent variables	–1	0	+1
X1	zein concentration (% m/V)	1.0	1.5	2.0
X2	hyaluronic acid concentration (% m/V)	0.1	0.2	0.3
X3	sodium caseinate concentration (% m/V)	0.5	1.25	2.0
X4	O/W ratio (v/v)	1:2	1:4	1:6

	dependent variables
R1	mean particle size
R2	polydispersity index
R3	zeta potential
R4	encapsulation efficiency

### 2.3 Preparation of Zein Nanoparticles

Nanoparticles were synthesized using the nanoprecipitation method, as described by Reboredo et al.^23^ The process involved the preparation of an aqueous phase and an organic phase. For the aqueous phase, HA and sodium caseinate were dissolved in ultrapure water under magnetic stirring. The organic phase was prepared by dissolving zein in an 80% hydroethanolic solution under continuous stirring. Subsequently, insulin (hydrophilic model drug) was added to the organic phase with stirring maintained. The organic phase was poured into the aqueous phase under constant agitation, resulting in nanoparticle formation. The organic solvent was then evaporated, and the resulting suspension was centrifuged at 16,500 rpm for 30 min at 20 °C. The nanoparticle pellet was resuspended in 1 mL of ultrapure water and stored at 2–8 °C for further analysis.

### 2.4 Physicochemical Characterization

#### 2.4.1 PS and PDI

The mean diameters of the nanoparticles and PDI were measured using dynamic light scattering with a Brookhaven 90 Plus instrument. Nanoparticles were diluted 1:100 (v/v) in ultrapure water and placed in a polystyrene cuvette for analysis. Measurements were performed at 25 °C, a scattering angle of 90°, and a laser wavelength of 660 nm. Each sample was analyzed in triplicate, and the results were expressed as the mean ± standard deviation.

#### 2.4.2 Zeta Potential

The ZP was determined via electrophoretic mobility measurements using a Malvern ZetaSizer ZS. Samples were diluted 1:100 (v/v) in a 1 mM KCl solution, placed in a DTS 1070 capillary cell, and analyzed at 25 °C with an applied potential of ± 150 mV. Triplicate measurements were performed, and the results were reported as the mean ± standard deviation.

#### 2.4.3 Morphology

The morphology of NP-Z was assessed by using a scanning electron microscope (SEM-TESCAN). A drop of nanoparticle dispersion was placed on a metal stub, dried at room temperature, and gold-coated under a vacuum for analysis.

#### 2.4.4 Encapsulation Efficiency (EE)

The EE of insulin (hydrophilic drug model) in nanoparticles was indirectly determined by using high-performance liquid chromatography (HPLC). A Waters 2695 Alliance HPLC system equipped with a 2998 photodiode array detector was employed. The chromatographic analysis utilized a C18 column (Atlantis T3 Waters, 250 × 4.6 mm, 5 μm) at 30 °C with ultrapure water acidified with 0.5% formic acid and acetonitrile (60:40, v/v) as the mobile phase, a flow rate of 1.0 mL/min, an injection volume of 20 μL, and detection at 272 nm. For further clarification, the validation of the analytical method performed is provided in the Supporting Information, demonstrating the adequacy of the method for the detection and quantification of insulin in the nanoparticle formulation, as well as its chromatogram. The chromatographic profile confirms the retention time and peak purity, ensuring that the insulin was accurately quantified.

The EE was calculated based on the initial amount of drug added (Di) and the free drug in the supernatant (Df) after centrifugation, using eq 1:
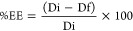
1

### 2.5 Evaluation of Nanoparticle Stability

The stability of the nanoparticles was evaluated in three batches stored at room temperature and under refrigeration (8 °C). Over 4 months, the drug content, PS, PDI, and ZP were monitored monthly. The drug content was quantified by HPLC, while PS, PDI, and ZP measurements were performed over 180 days.

### 2.6 In Vitro Release

An in vitro release study was conducted in triplicate using a shaker followed by centrifugation.^24^ NPs containing INS were incubated in a PBS buffer solution (pH 7.4) at 37 °C with agitation at 150 rpm. Samples were collected at predefined intervals for up to 96 h, centrifuged, and filtered. The released INS content was quantified by HPLC. Release kinetics were analyzed using KinetDS software, applying various mathematical models, including the zero-order, first-order, second-order, third-order, Higuchi model, Weibull model, Hixson–Crowell model, Baker–Lonsdale model, and Korsmeyer–Peppas model.^25^

### 2.7 Statistical Analysis

Data were presented as the mean ± standard deviation and analyzed using Minitab software. Statistical significance was determined at *p* < 0.05.

## 3 Results and Discussion

### 3.1 Optimization of Zein Nanoparticle Preparation via Nanoprecipitation Using a 2^4^ Factorial Design

To evaluate the influence of formulation and process variables on nanoparticle properties, a total of 22 experiments were conducted, including six central points to estimate the pure error and assess the precision of the experimental model. The primary response variables analyzed were average particle diameter (R1), PDI (R2), ZP (R3), and %EE (R4). The experiments were designed to capture the effects of the independent variables while ensuring reproducibility and accuracy. The detailed results of all experiments are presented in Table 2, providing a comprehensive overview of the response variables under different experimental conditions.

**Table 2:** Results of Dependent Variables for the Optimization of Zein Nanoparticle Production Using the Nanoprecipitation Method^a^

formulation	R1	R2	R3	R4
F1	161 ± 4	0.130 ± 0.030	–22 ± 3.0	20 ± 6
F2	180 ± 2	0.130 ± 0.015	–24 ± 1.0	19 ± 2
F3	233 ± 11	0.116 ± 0.011	–31 ± 1.0	30 ± 3
F4	188 ± 8	0.130 ± 0.024	–23 ± 2.0	22 ± 2
F5	200 ± 4	0.143 ± 0.011	–24 ± 3.0	18 ± 5
F6	181 ± 2	0.247 ± 0.013	–19 ± 2.0	20 ± 2
F7	235 ± 7	0.121 ± 0.028	–27 ± 2.0	15 ± 2
F1	177 ± 5	0.134 ± 0.030	–25 ± 2.0	19 ± 1
F1	160 ± 2	0.143 ± 0.008	–23 ± 2.0	18 ± 2
F8	193 ± 3	0.128 ± 0.034	–28 ± 2.0	20 ± 5
F9	155 ± 6	0.159 ± 0.035	–23 ± 1.0	18 ± 3
F10	192 ± 1	0.132 ± 0.013	–26 ± 2.0	20 ± 5
F1	189 ± 8	0.171 ± 0.014	–17 ± 0.4	20 ± 4
F1	174 ± 2	0.149 ± 0.019	–24 ± 1.0	15 ± 6
F11	196 ± 8	0.163 ± 0.015	–26 ± 0.4	18 ± 4
F12	194 ± 5	0.121 ± 0.026	–23 ± 6.0	20 ± 5
F13	235 ± 2	0.126 ± 0.016	–30 ± 2.0	16 ± 6
F14	245 ± 7	0.105 ± 0.018	–32 ± 2.0	18 ± 2
F15	198 ± 4	0.151 ± 0.041	–19 ± 3.0	18 ± 2
F1	155 ± 1	0.153 ± 0.011	–25 ± 2.0	22 ± 4
F16	147 ± 5	0.171 ± 0.034	–20 ± 2.0	17 ± 2
F17	176 ± 4	0.236 ± 0.010	–17 ± 2.0	16 ± 2

a The dependent variables, also referred to as response variables, are represented by R1 (mean diameter), R2 (PDI), R3 (ZP), and R4 (%EE), presented as the mean ± standard deviation (*n* = 3).

Statistical analyses, including multiple regression, ANOVA, and significance testing (*F*-value and *p*-value), were performed to validate the model and quantify the impact of each factor on the response variables. The residual plots and histograms for PS, PDI, and ZP confirm the reliability of the factorial design in predicting the outcomes (see the Supporting Information). These analyses demonstrated that the regression models provided a reliable fit to the experimental data with all significant effects identified. The results for each response variable are presented and discussed individually, offering a detailed understanding of how different factors influence the physicochemical properties of zein nanoparticles.

#### 3.1.1 PS and Influencing Factors

**Figure 1:**
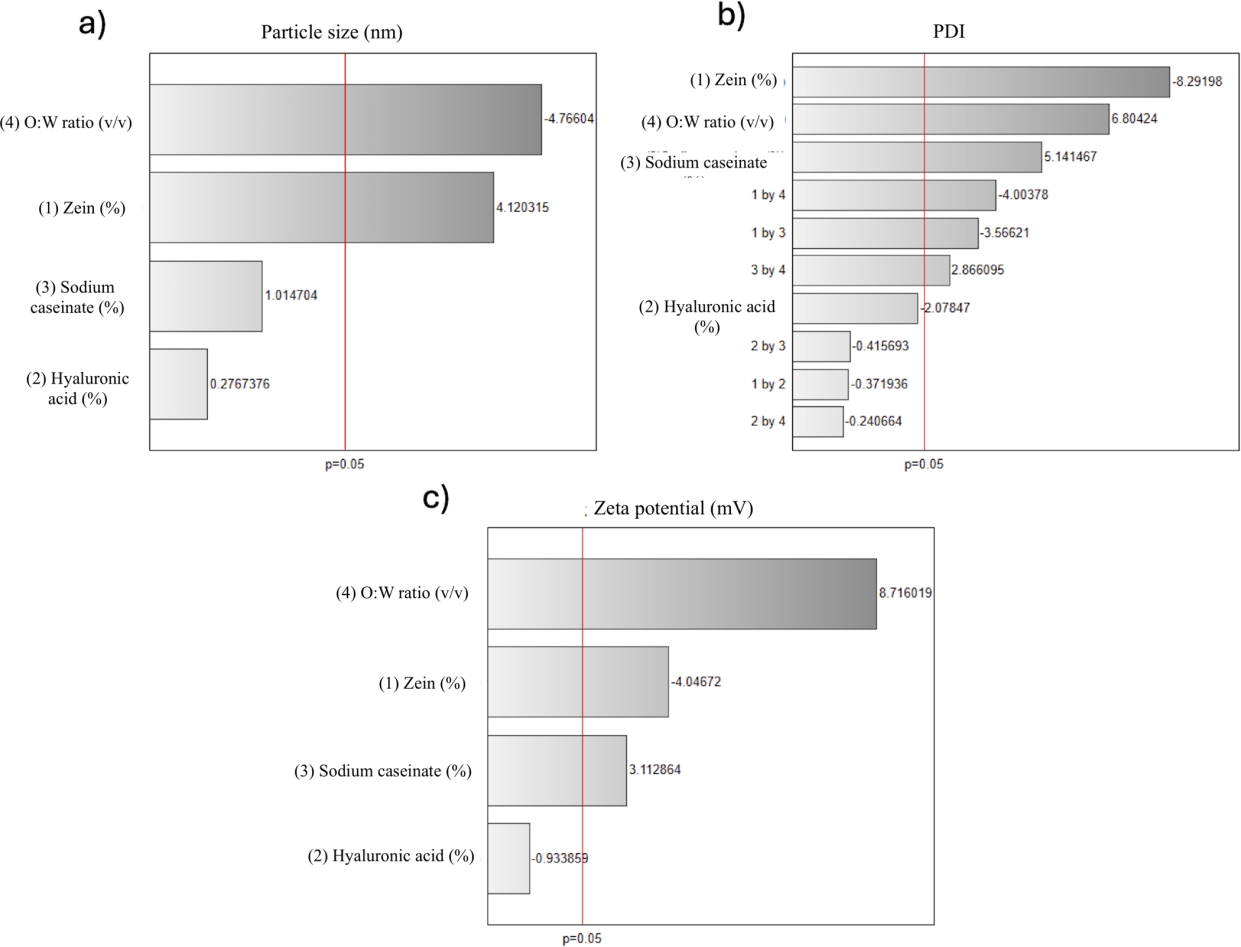
Pareto charts showing the influence of formulation factors on (a) PS, (b) PDI, and (c) ZP.

The Pareto chart for PS (Figure 1a) highlights that the O/W ratio exerts the most significant negative effect, where higher ratios result in smaller nanoparticles. This likely reflects enhanced molecular dispersion in the aqueous phase, promoting the formation of smaller particles. In contrast, zein concentration showed the most prominent positive effect, with higher concentrations leading to larger PSs, possibly due to increased solution viscosity, which can hinder particle breakup during nanoprecipitation and facilitate aggregation.^11,24^ Sodium caseinate and HA had smaller impacts on PS, indicating their primary role lies in stabilization rather than size determination.^25,26^

**Figure 2:**
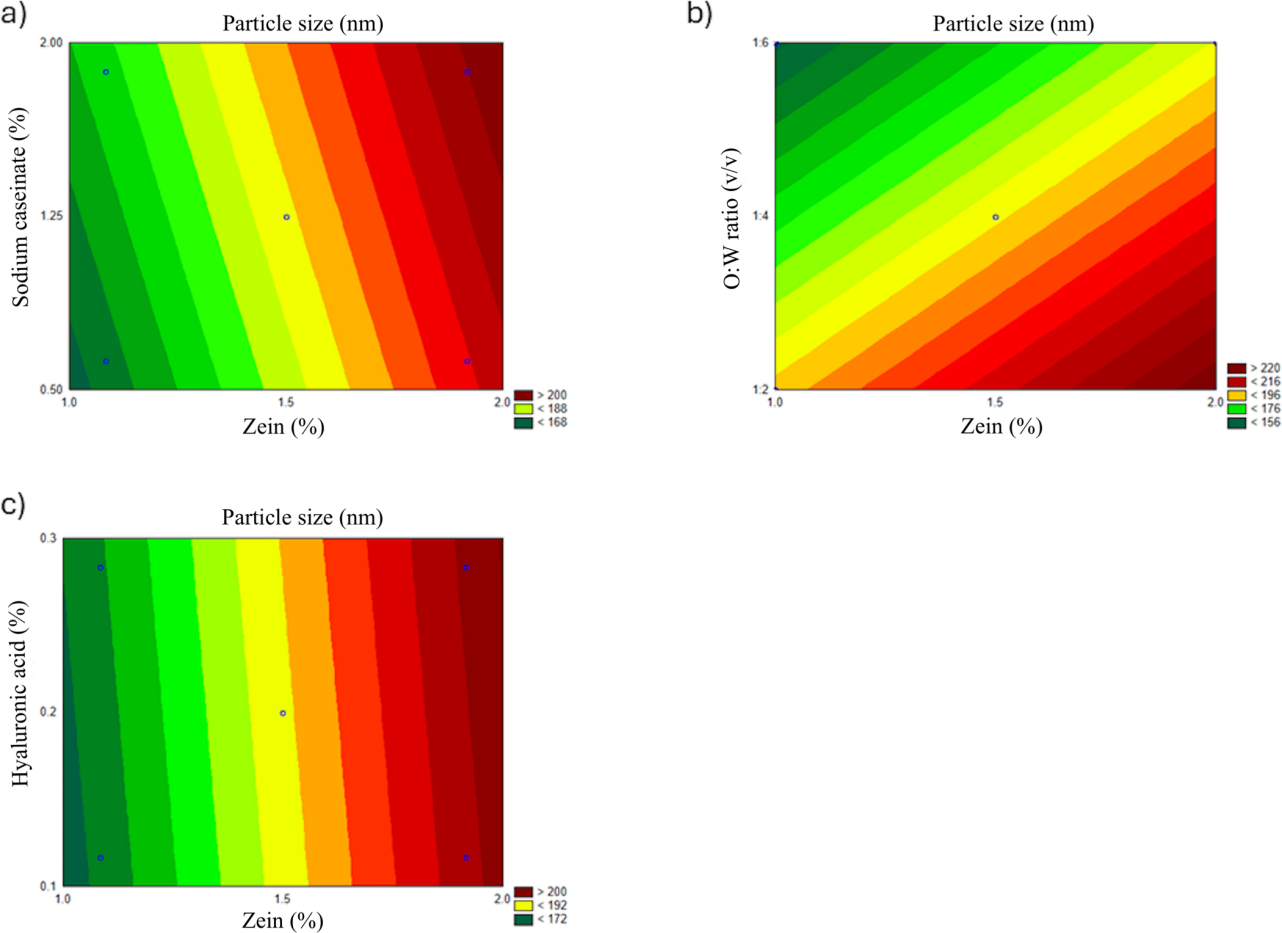
Contour plots illustrating the interaction effects of formulation factors on PS: (a) zein concentration vs sodium caseinate, (b) zein concentration vs O/W ratio, and (c) zein concentration vs HA.

The contour plot for the interaction between the O/W ratio and zein concentration (Figure 2b) further illustrates these effects. Smaller PSs are achieved when the O/W ratio is increased and the zein concentration is kept low. Conversely, higher zein concentrations combined with lower O/W ratios produce larger particles, underscoring the importance of balancing these two factors. The interaction of zein concentration with sodium caseinate (Figure 2a) shows that while sodium caseinate plays a stabilizing role, its influence on PS is secondary to that of zein. Similarly, HA (Figure 2c) has a minimal impact on PS as its primary role lies in stabilization rather than size modulation. These interactions suggest that optimizing the zein concentration and O/W ratio is critical for controlling PS.

PS is a critical parameter in the design of nanoparticles for oral administration. Smaller particles, typically below 300 nm, are more readily absorbed in the gastrointestinal tract due to their ability to traverse mucus layers and interact with intestinal epithelial cells. This size range ensures better bioavailability of the encapsulated active compounds and can also protect the drug from enzymatic degradation. Additionally, smaller particles offer improved drug distribution, reduced variability in absorption, and enhanced therapeutic efficacy.^27−29^ Thus, understanding and controlling PS are essential for optimizing nanoparticle-based delivery systems for oral applications.

#### 3.1.2 PDI and Homogeneity

Optimizing PDI is crucial in nanoparticle formulation as a lower PDI indicates a more uniform PS distribution, enhancing stability and performance. A PDI value below 0.3 is generally considered acceptable for ensuring homogeneity in nanoparticle preparations.^30^ The Pareto chart (Figure 1b) reveals that the zein concentration had the strongest negative effect on PDI, suggesting that higher concentrations promote more uniform nanoparticles. The O/W ratio, in contrast, had a positive effect, indicating that higher ratios can lead to broader PS distributions. Sodium caseinate also showed a moderate positive effect on PDI, reflecting its role as a stabilizer that can sometimes introduce slight variability.

**Figure 3:**
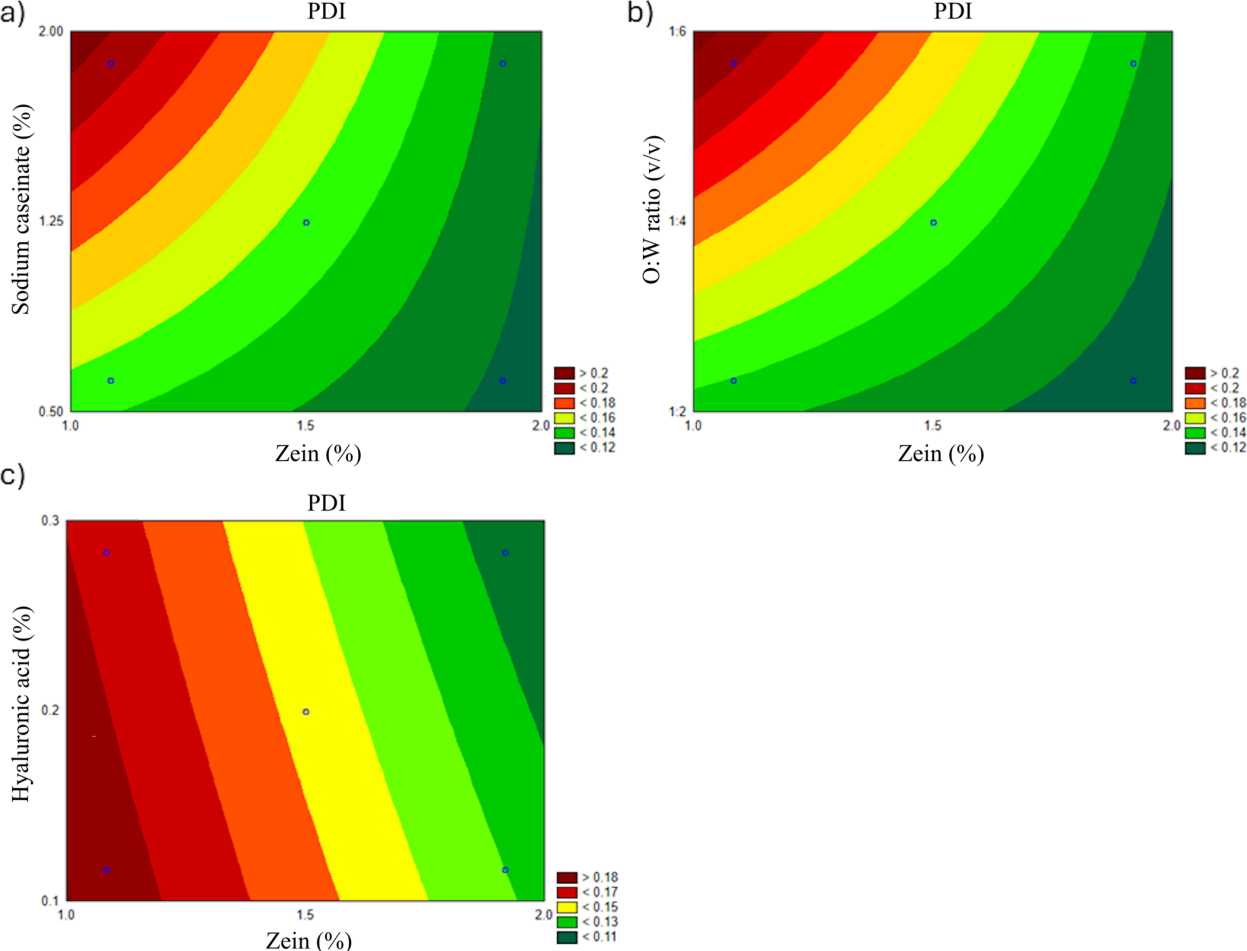
Contour plots illustrating the interaction effects of formulation factors on PDI: (a) zein concentration vs sodium caseinate, (b) zein concentration vs O/W ratio, and (c) zein concentration vs HA.

The contour plots support these findings (Figure 3). Lower zein concentrations combined with higher O/W ratios yield the highest PDI values, reflecting less uniform particle populations (Figure 3b). On the other hand, higher zein concentrations consistently produce lower PDI values, signifying improved uniformity (Figure 3a,c). Sodium caseinate and HA play complementary roles in improving particle homogeneity under specific conditions.

#### 3.1.3 ZP and Stability

The Pareto chart (Figure 1c) shows that the O/W ratio has the most significant positive effect on ZP, suggesting that increasing the ratio enhances the particle surface charge and stability. Zein concentration had a negative effect, likely due to changes in particle surface composition with a higher zein content. Sodium caseinate positively influenced ZP, emphasizing its role as a stabilizer that enhances surface charge, while HA showed minimal direct impact. However, despite its limited effect on ZP, HA was included in the formulation primarily for its mucoadhesive properties rather than its role in colloidal stabilization. Its ability to interact with mucins in the gastrointestinal tract improves the retention of nanoparticles at the absorption site, enhancing drug bioavailability.^26,27^ Importantly, the optimized HA concentration did not significantly increase the formulation viscosity, ensuring ease of handling while providing additional therapeutic benefits.

**Figure 4:**
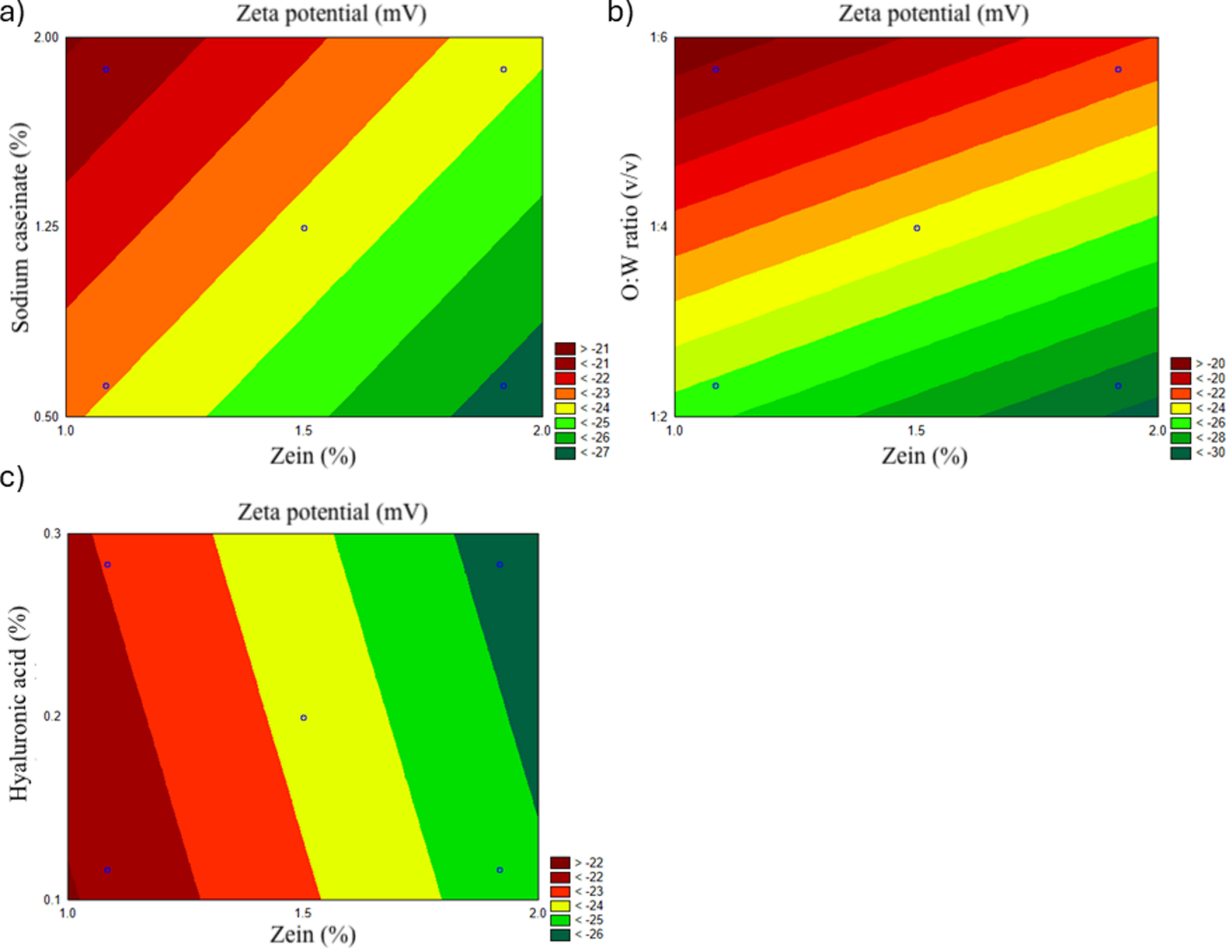
Contour plots illustrating the interaction effects of formulation factors on PDI: (a) zein concentration vs sodium caseinate, (b) zein concentration vs O/W ratio, and (c) zein concentration vs HA.

The contour plot (Figure 4b) illustrates that higher O/W ratios result in the most stable particles, as indicated by higher ZP values. Moderate zein concentrations further enhance stability, while very high zein levels reduce the ZP. Sodium caseinate plays a complementary role in stabilizing nanoparticles (Figure 4a), while HA has negligible influence on ZP (Figure 4c).

ZP is a critical parameter in nanoparticle formulation as it reflects the surface charge of particles and significantly influences colloidal stability. Particles with high ZP values (either positive or negative) tend to repel each other, preventing aggregation and ensuring a stable suspension. Conversely, low ZP values can lead to particle aggregation due to insufficient repulsive forces, resulting in instability. Beyond stability, the ZP plays a pivotal role in the interaction of nanoparticles with cellular membranes and biological surfaces. Positively charged nanoparticles can interact more effectively with the negatively charged cell membranes, enhancing cellular uptake, while negatively charged or neutral particles may exhibit reduced clearance and prolonged circulation times in biological systems.^31−33^ These interactions highlight the importance of optimizing the ZP not only for colloidal stability but also for achieving the desired biological performance in drug delivery systems.

The enhanced stability, size distribution, and ZP of the zein nanoparticles can be attributed to the complementary roles of sodium caseinate and HA. Sodium caseinate acts primarily as a steric stabilizer, adsorbing onto the nanoparticle surface and providing a protective barrier that prevents aggregation by steric hindrance.^28,29^ Additionally, the negative charges of sodium caseinate contribute to the overall surface charge, enhancing electrostatic repulsion between particles, which is reflected in the improved ZP values observed. On the other hand, HA, despite having minimal direct impact on ZP, contributes to stability through its hydrophilic nature, which enhances the hydration layer around the nanoparticles, reducing the likelihood of particle coalescence. The mucoadhesive properties of HA also facilitate prolonged retention in biological environments, which can indirectly influence the stability and size distribution of the nanoparticles.^27,30^ These synergistic effects help to maintain colloidal stability and uniformity in PS, making the zein-caseinate-HA nanoparticles a robust system for drug delivery applications.

#### 3.1.4 Encapsulation Efficiency

The analysis of variance (ANOVA) for the EE of insulin in the nanoparticles revealed that the applied linear model was insufficient to explain the observed variability. This limitation was evident from the low coefficient of determination (*R*^2^ = 18.89%), indicating that the selected factors accounted for only a small fraction of the variation in the EE. As a result, surface plots and Pareto charts were not generated, as they would lack statistical significance and could lead to unreliable interpretations of the effects of the studied variables. These findings suggest that EE is likely influenced by additional factors or complex interactions not captured by the current model, highlighting the need for further investigation and potentially more advanced experimental designs to fully understand and optimize this parameter.

According to Table 2, the EE of insulin within zein nanoparticles ranged from 15 to 30%. This relatively modest EE can be attributed to several factors inherent to the formulation process and the physicochemical properties of both insulin and zein. Insulin, being a hydrophilic peptide, exhibits limited affinity for the hydrophobic domains of zein proteins, which can hinder effective encapsulation. Additionally, the nanoprecipitation method employed may not facilitate optimal interactions between insulin and zein, leading to partial encapsulation.^28^

One of the main limitations of the current experimental design is the use of zein, a hydrophobic protein, as the primary matrix material. This characteristic naturally limits the interaction with hydrophilic molecules, such as insulin, leading to partial drug partitioning into the aqueous phase during the nanoprecipitation process. Additionally, the lack of hydrophilic modifications to the zein matrix may have further restricted the EE. To address these limitations in future studies, several alternative strategies can be considered. The incorporation of hydrophilic modifiers could enhance the compatibility between insulin and the zein matrix, improving the EE. Surface modification of zein through chemical modifications to introduce hydrophilic functional groups could strengthen interactions between insulin and the polymer, enhancing drug retention within the nanoparticles. Moreover, coencapsulation strategies, such as the inclusion of surfactants or secondary polymers during the nanoprecipitation process, might help retain insulin within the hydrophobic matrix, minimizing drug loss to the aqueous phase. Additionally, precomplexation of insulin with polyelectrolytes prior to encapsulation could reduce the hydrophilicity of insulin, thereby improving its retention within the zein matrix. These strategies could potentially enhance the EE of insulin while maintaining the stability and functionality of the nanoparticles for oral delivery applications.

The overall findings demonstrate that the O/W ratio and zein concentration are the most influential factors in determining PS, PDI, and ZP. The O/W ratio is critical for achieving smaller particles with higher stability, making it a key parameter for oral delivery systems. Zein concentration affects both PS and uniformity, highlighting the need for precise control to optimize the nanoparticle performance. Stabilizers such as sodium caseinate enhance colloidal stability and uniformity, while HA plays a supportive role in maintaining particle dispersion.

Model validation through residual analysis confirms the robustness and accuracy of the experimental design, ensuring that the observed relationships are reliable and reproducible. These insights provide a solid foundation for optimizing zein nanoparticle formulations for oral drug delivery, where precise control of the size, uniformity, and stability is critical for improving therapeutic outcomes.

#### 3.1.5 Multivariate Analysis: Hierarchical Cluster Analysis (HCA) and Principal Component Analysis (PCA)

**Figure 5:**
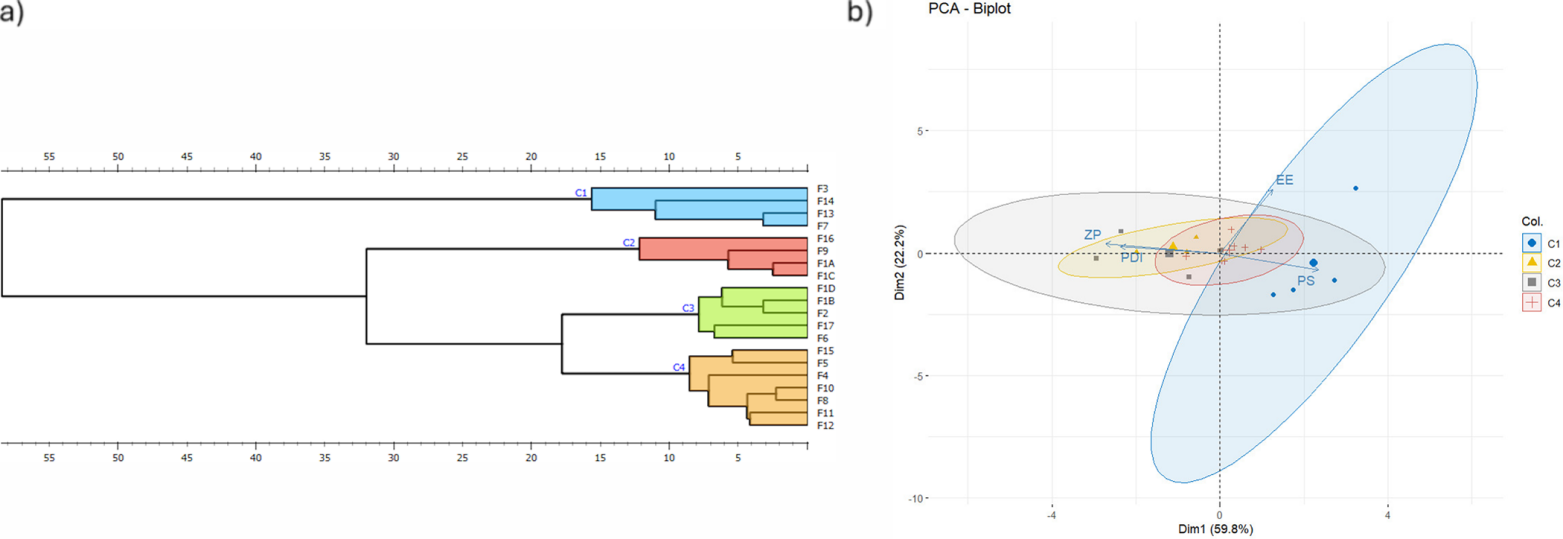
(a) HCA dendrogram and (b) PCA biplot for the evaluation of zein nanoparticle formulations.

The HCA dendrogram (Figure 5a) and PCA biplot (Figure 5b) were used to explore relationships among the formulation parameters and response variables, offering a comprehensive understanding of the data structure and grouping trends.

The HCA dendrogram categorizes the formulations into four distinct clusters (C1–C4) based on similarities in PS, PDI, ZP, and EE. Cluster C1 predominantly includes formulations with smaller PSs and higher stability (indicated by high ZP), reflecting optimized conditions for the nanoparticle properties. C2 and C3 include intermediate formulations with moderate PSs and PDI values, while C4 comprises formulations with larger PSs and higher variability, often associated with suboptimal conditions. The clustering pattern underscores the critical influence of the O/W ratio and zein concentration in determining nanoparticle properties, as formulations with similar levels of these factors tend to group together.

The PCA biplot further corroborates these findings, with the first two principal components Dim1 and Dim2 explaining 59.8 and 22.2% of the total variance, respectively. PS is strongly aligned along Dim1, indicating its dominant role in distinguishing formulations, while PDI and the ZP contribute more to Dim2. EE, however, shows a less pronounced alignment with either component, reflecting the weak explanatory power of the current model for this parameter, as previously discussed.

Clusters identified in the PCA biplot align well with those from HCA, with formulations in C1 occupying the region associated with smaller PSs, higher ZP, and lower PDI. Formulations in C4, in contrast, are positioned toward larger PSs and higher PDI, reflecting suboptimal stability and uniformity. C2 and C3 represent transitional clusters with overlapping characteristics, indicating intermediate levels of optimization.

The integration of HCA and PCA provides valuable insights into the relationships between formulation parameters and response variables. The grouping observed in HCA highlights the importance of key variables, such as the O/W ratio and zein concentration, in driving the clustering of formulations with favorable PS and stability. Smaller PSs and lower PDI values observed in C1 align with the requirements for oral drug delivery as these properties enhance bioavailability, ensure consistent drug release, and minimize variability in therapeutic outcomes.

The PCA biplot emphasizes the strong influence of PS on the overall variance in the data set, confirming its critical role in nanoparticle performance. The alignment of PDI and ZP with Dim2 highlights their importance in stability and homogeneity, which are essential for maintaining colloidal integrity during storage and administration. The weaker contribution of EE in the PCA suggests that factors outside the current experimental scope may play a significant role in the EE, warranting further investigation.

These multivariate analyses complement the individual response evaluations, offering a holistic perspective on how formulation parameters interact to influence nanoparticle properties. By identifying clusters and principal contributors, the study highlights potential strategies for optimizing formulations tailored for oral administration, focusing on achieving the desired balance among PS, stability, and EE.

### 3.2 Response Optimization

**Figure 6:**
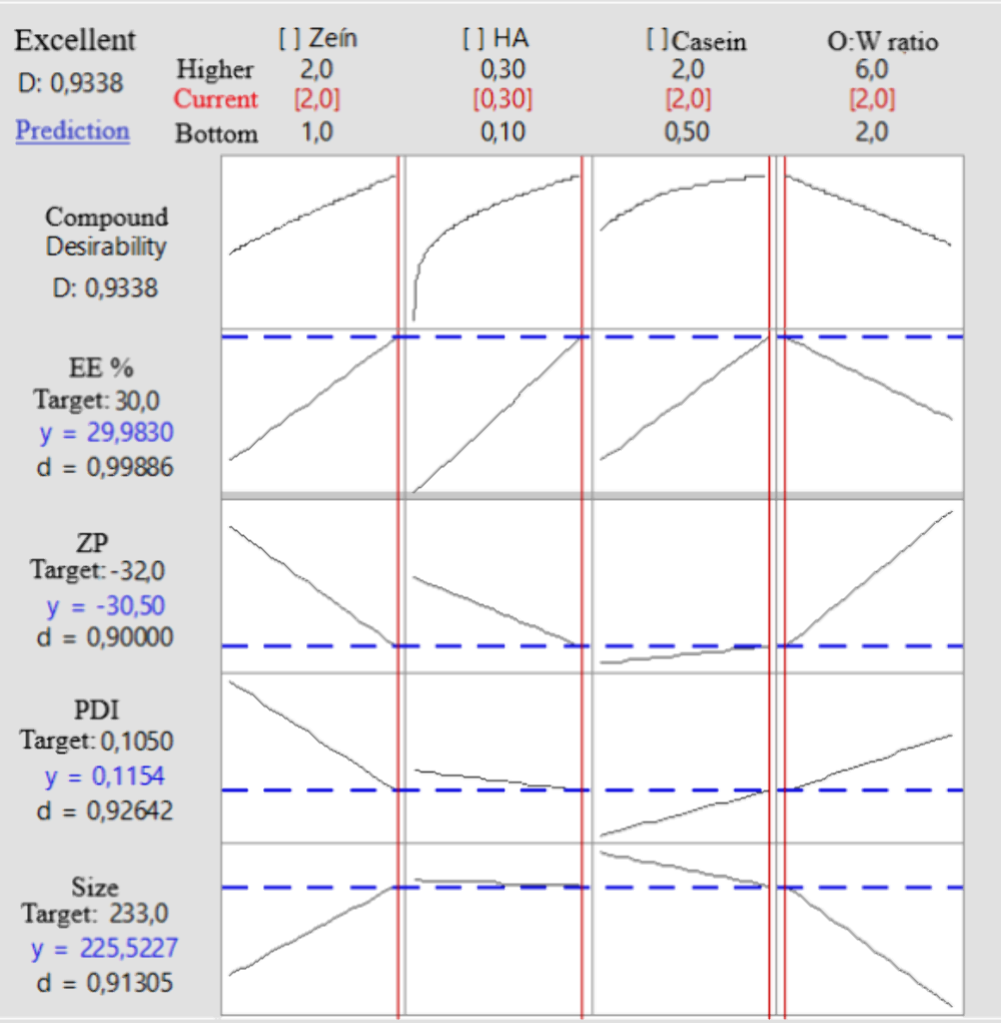
Response optimization plot for zein nanoparticle formulations, highlighting compound desirability and individual response predictions.

The response optimization chart (Figure 6) summarizes the influence of formulation parameters—zein concentration, HA concentration, sodium caseinate concentration, and O/W ratio—on multiple response variables, including encapsulation efficiency (EE%), ZP, PDI, and PS. The desirability function (*D* = 0.9338) reflects an excellent overall fit, indicating that the optimal combination of variables successfully meets the target values for all responses (Table 3).

**Table 3:** Optimization Targets for Zein Nanoparticle Parameters

parameter	goal	target
particle size	target	233 nm
PDI	target	0.100
zeta potential	target	–32 mV
%EE	target	30

The optimization plot reveals that EE is maximized at higher zein concentrations (2.0%) and sodium caseinate concentrations (2.0%), while the ratio of encapsulation to water (2:6) has a lesser impact. The EE target of 30.0% is nearly achieved, with a predicted value of 29.9830% (*d* = 0.99886). This suggests that zein and caseinate play critical roles in encapsulating insulin, likely due to their ability to stabilize and retain the hydrophilic drug within the nanoparticle matrix. HA concentration has a minimal effect on EE, consistent with its role as a dispersant rather than an encapsulation enhancer.

The ZP is strongly influenced by the O/W ratio, with higher ratios contributing to a surface charge close to the target of −32.0 mV. The predicted value of −30.50 mV (*d* = 0.9000) indicates satisfactory colloidal stability, as values above ±30 mV are typically associated with stable dispersions. Zein and caseinate concentrations have a secondary role, with slight improvements in stability observed at higher levels. HA concentration exerts a minimal influence on ZP.

For PDI, the optimization chart shows that higher sodium caseinate concentrations (2.0%) and moderate HA levels (0.30%) contribute to achieving a low, uniform size distribution. The target PDI of 0.1050 is slightly exceeded, with a predicted value of 0.1154 (*d* = 0.92642). While the O/W ratio has a moderate effect, zein concentration exhibits a limited impact, reflecting its primary influence on PS rather than uniformity.

The PS target of 233.0 nm is closely achieved, with a predicted value of 225.5227 nm (*d* = 0.91305). The O/W ratio is the most significant determinant, with higher ratios leading to smaller PSs. Zein concentration also plays an important role, with higher levels contributing to larger particles. Sodium caseinate and HA concentrations exhibit limited effects on size, reinforcing their primary roles as stabilizers rather than size modulators.

The optimization results provide a comprehensive understanding of how formulation parameters interact to achieve the desired nanoparticle characteristics. The high desirability score (*D* = 0.9338) confirms that the optimal conditions—zein at 2.0%, HA at 0.30%, sodium caseinate at 2.0%, and the O/W ratio between 2:6—balance EE, stability, size uniformity, and PS within acceptable ranges. These conditions highlight the critical role of zein and caseinate in stabilizing the formulation and encapsulating the drug, while the O/W ratio predominantly controls PS and ZP.

The close alignment of predicted and target values across all responses underscores the robustness of the optimization process and its applicability for tailoring nanoparticle formulations for oral delivery. By fine-tuning these parameters, it is possible to achieve nanoparticles with ideal physicochemical properties, enhancing bioavailability and therapeutic performance. These findings provide a strong foundation for future scale-up and applications in drug delivery systems.

**Figure 7:**
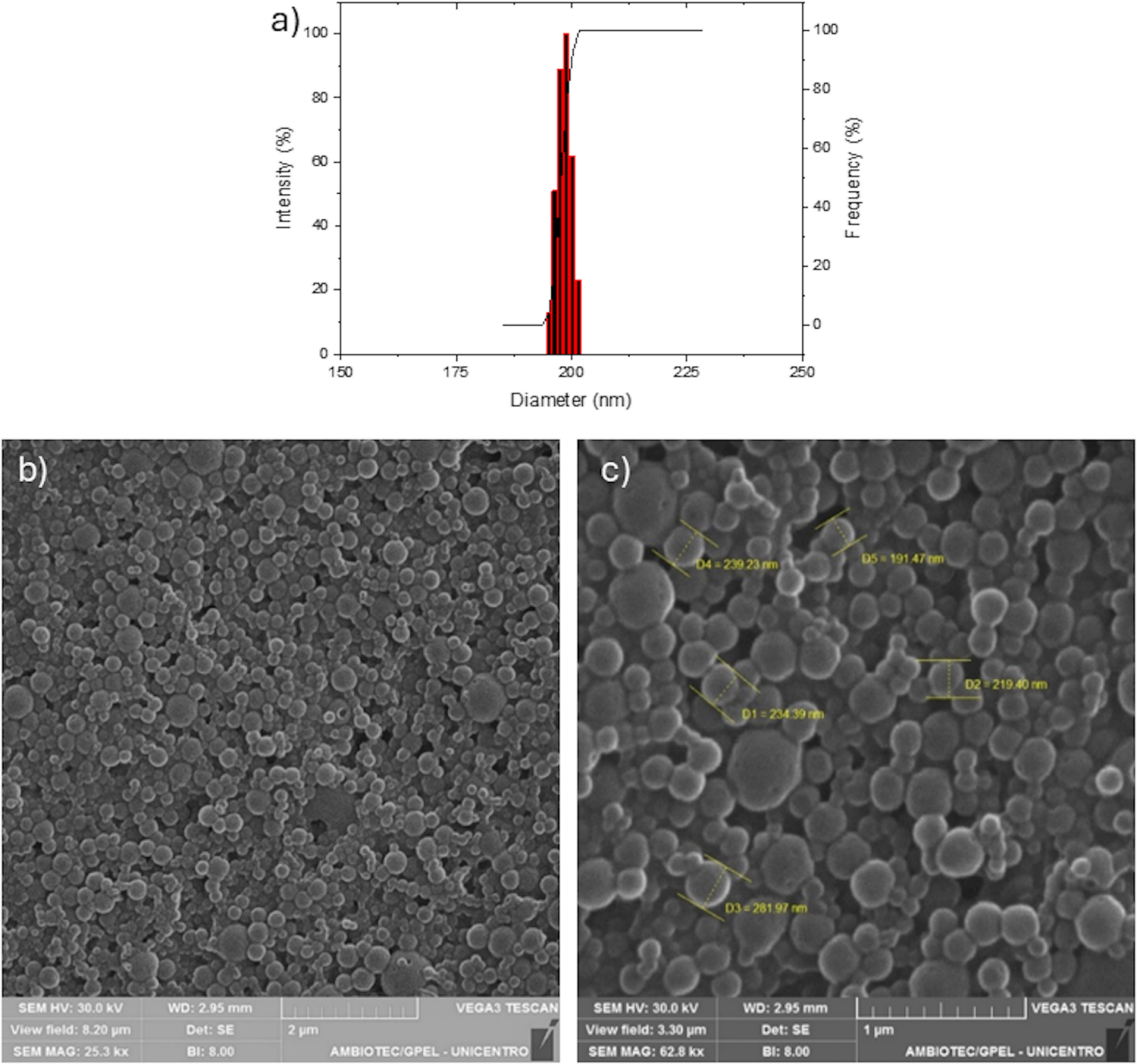
Characterization of optimized zein nanoparticles: (a) PS distribution, (b) SEM image of nanoparticles at low magnification, and (c) SEM image of nanoparticles at high magnification with size measurements.

After the optimization process, the optimized nanoparticles exhibited a PS of 217.3 ± 5.4 nm, a PDI of 0.077 ± 0.022, a ZP of −24.7 ± 1.9 mV, and an EE of 31% ± 4. The nanoparticles displayed a monomodal size distribution (Figure 7a) and a spherical morphology, as confirmed by SEM images (Figure 7b,c).

These results highlight the success of the optimization process in producing zein-based nanoparticles with favorable physicochemical characteristics for oral administration. The combination of small PS, low polydispersity, adequate ZP, and efficient encapsulation ensures enhanced stability, bioavailability, and uniformity, which are critical for achieving consistent therapeutic outcomes. Furthermore, the monomodal size distribution and spherical morphology reinforce the suitability of these nanoparticles for controlled drug delivery applications.

### 3.3 Evaluation of Stability

**Figure 8:**
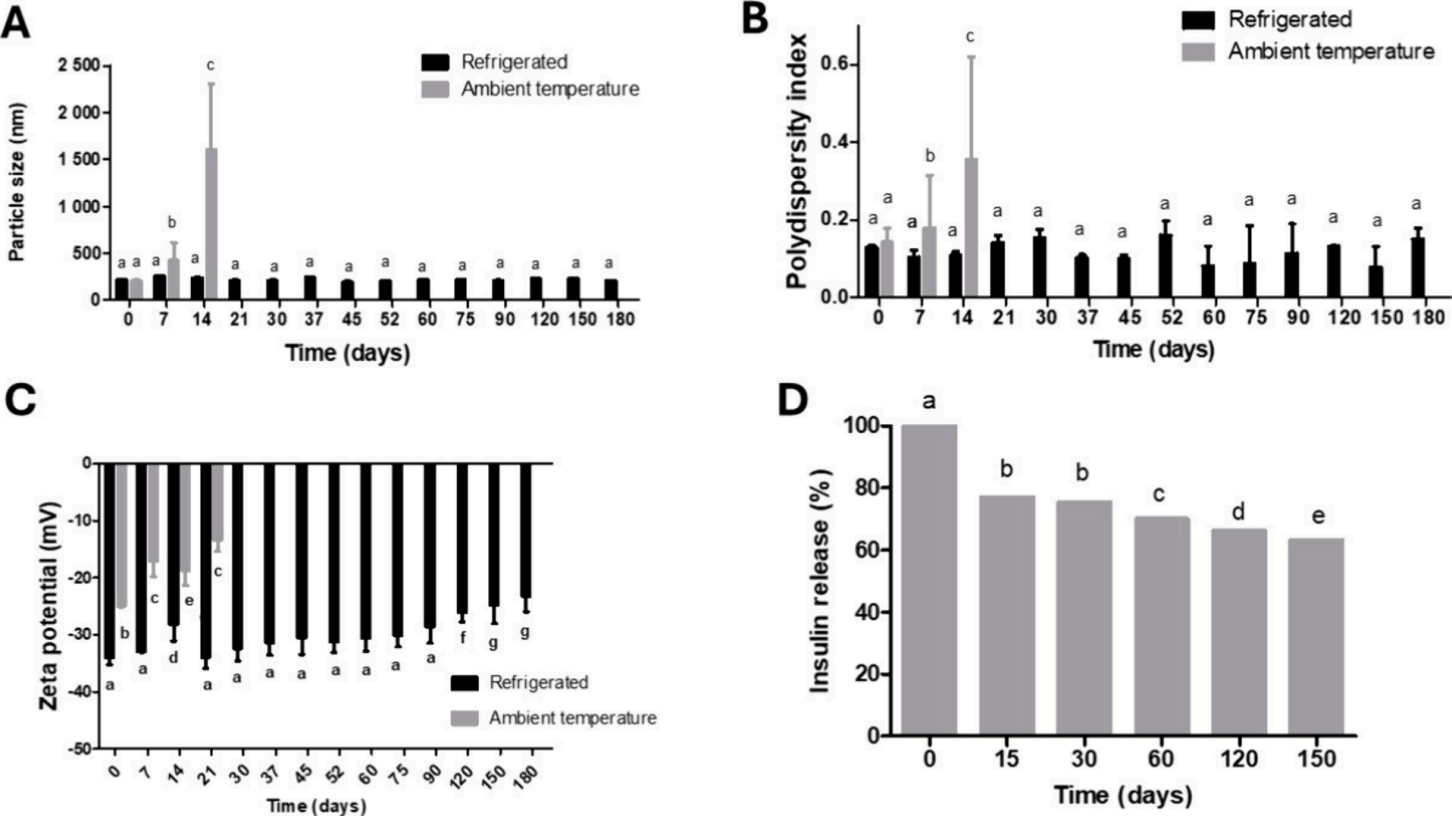
Nanoparticles under different storage conditions (*n* = 3). PS (A), PDI (B), ZP (C), and drug content (D). a, b, c, d, e Same letters indicate statistical equality, and different letters indicate statistical inequality (two-way ANOVA with Bonferroni’s post-test and α < 0.05).

The stability of NPs is a fundamental factor in the development of drug delivery systems as their instability can compromise the efficacy and safety of the product over time.^31^ Parameters such as PS, PDI, ZP, and drug content were monitored for 180 days under two storage conditions: room temperature (25 °C) and refrigeration (8 °C). The results of these analyses are presented in Figure 8.

At room temperature, a significant increase in PS (Figure 8A) and PDI (Figure 8B) of the nanoparticles was observed during the first 14 days, indicating the formation of aggregates and a loss of stability, which led to the discontinuation of analyses under this storage condition. Under refrigeration, the parameters PS, PDI, and ZP (Figure 8A–C) remained stable, indicating that a temperature of 8 °C preserves the integrity and electrostatic stability of the NPs, corroborating the findings of Chen et al., who also observed the stability of zein NPs with HA under refrigeration (8 °C) for up to 8 months.^32^

Observing the ZP results in Figure 8C, it is evident that nanoparticles stored at room temperature (gray bars) lose their negative charge significantly faster compared to those stored under refrigeration (black bars). This loss of negative charge may be associated with a decrease in the system stability, justifying the discontinuation of subsequent analyses for this storage condition.

Under refrigeration, however, the ZP of the NPs remained more negative, with values ranging from −34 mV at the beginning to −23 mV at the end of 180 days. A high ZP value (typically above ± 30 mV) indicates a stable dispersion, where particles have a lower tendency to agglomerate, preventing the formation of aggregates that could impair the properties of the NPs.^33,34^

Due to the instability of NPs stored at room temperature, the EE analysis was performed only for samples maintained under refrigeration, as shown in Figure 8D.

Nevertheless, even under refrigeration, there was a significant reduction in the encapsulated insulin concentration, with losses of 23% in 15 days and up to 37% after 150 days, indicating possible degradation or premature release of the drug. This initial release may be related to the presence of drug molecules adsorbed on the surface of the NPs or differences in the solubility of the encapsulation material.^35−37^

To overcome this issue, future studies could explore alternative storage conditions, such as lower temperatures, and/or examine the interactions between INS and the matrix components of the NPs, aiming to better understand degradation and improve system stability.

In summary, the stability of the nanoparticles is preserved under refrigeration (8 °C), as evidenced by the maintenance of PS, PDI, and ZP over 180 days. These results indicate that refrigeration prevents the formation of aggregates and ensures the uniformity and electrostatic stability of the system. However, a significant reduction in the encapsulated INS concentration was observed over time, with progressive losses that could compromise therapeutic efficacy in prolonged periods.

Although storage at 8 °C is effective in stabilizing the physical properties of the NPs, improvements are necessary to prevent degradation or premature release of INS. Future studies should explore alternative storage conditions, such as lower temperatures, and/or investigate the interactions between the matrix components and the drug to optimize the chemical and functional stability of the system.

### 3.4 In Vitro Release Study

**Figure 9:**
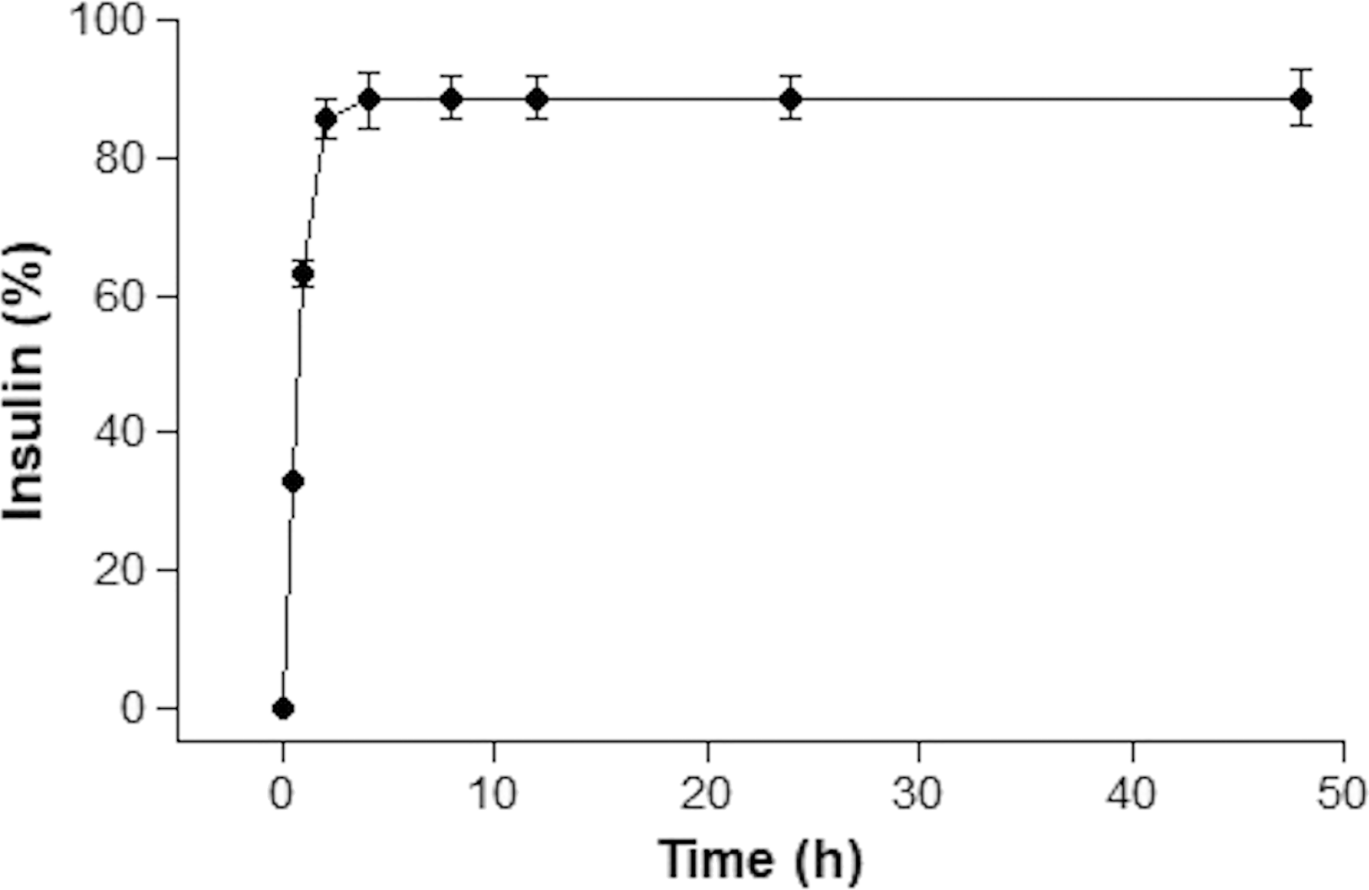
In vitro insulin release from nanoparticles in PBS solution (50 mM) pH 6.5 at 37 °C over 48 h (*n* = 3).

The release profile of INS in PBS (50 mM, pH 7.4) simulates the behavior of the nanoparticles under physiological conditions representative of blood plasma and is illustrated in Figure 9.

As observed in Figure 9, approximately 33% of the INS was released within the first 30 min, increasing to 63% in 1 h and reaching 85% in 2 h. It is important to note that due to the hydrophilic nature of INS and its affinity for water, its encapsulation within NPs presents a greater challenge, resulting in a tendency for faster release. This rapid initial release is significant in the case of INS as it enables an immediate therapeutic response, especially in situations of acute hyperglycemia, where a rapid reduction in blood glucose levels is crucial to prevent severe complications.^38,39^

After 2 h, the cumulative release reached 85%, with a moderate increase to 88% at 4 h. This behavior represents a transition to a more controlled and sustained release, characteristic of INS molecules encapsulated in the core of the NPs, which are gradually released. From 8 h onward, the cumulative release stabilized at 88.7%, remaining constant up to 48 h. This stabilization suggests that the release system reached its maximum capacity, releasing almost all of the encapsulated INS that was available.

To evaluate the release kinetics of INS from the NPs, the data obtained were mathematically analyzed using KinetDS software. The criterion used for model selection was the correlation coefficient (*r*), aiming to identify the kinetic model that best describes the drug release mechanism and to predict the process by which the release occurs. The applied mathematical models and their respective r values are presented in Table 4.

**Table 4:** Kinetic Analysis of Insulin Release from NPS in PBS Solution (50 MM, pH = 7.4) (*r* = Correlation Coefficient)

model	*r*
zero-order	0.1818
first-order	0.1595
second-order	0.1376
third-order	0.1195
Korsmeyer–Peppas	0.9252
Weibull	0.9159
Hixson–Crowell	0.1671
Higuchi	–9.1156
Michaelis–Menten	0.9459
Hill	0.6482

Among the tested models, the Michaelis–Menten model (*r* = 0.9459) showed the best fit to the experimental data, followed by the Korsmeyer–Peppas model with a lag time (*r* = 0.9252) and the Weibull model (*r* = 0.9159). These results indicate that the release of INS does not follow a purely diffusional pattern but rather a mechanism controlled by nonlinear processes, possibly related to the saturation of release sites in the polymeric matrix.^25^

In contrast, the zero-order, first-order, second-order, and third-order models showed low correlation coefficients (*r* < 0.2), suggesting that the release kinetics cannot be explained by simple models. The high correlation observed for the Michaelis–Menten model reinforces the hypothesis that the release occurs through a saturable mechanism, typical of systems where the release rate reaches a plateau due to limitations in matrix capacity or drug availability.^40,41^

The relevance of the fit to the Michaelis–Menten model may be associated with the physicochemical properties of the zein, sodium caseinate, and HA matrix, which control insulin diffusion and influence release through matrix hydration and swelling. Thus, the combination of these components appears to promote a controlled release, initially limited by diffusion and later affected by the saturation of the release sites.

## 4 Conclusions

The results of this study substantiate the effectiveness of the factorial experimental design as a reliable methodology for optimizing zein nanoparticle formulations. This approach enabled the identification and analysis of critical factors, including the mean diameter, PDI, ZP, and EE. This allowed for precise adjustments to be made in order to achieve the optimal nanoparticle properties. The findings indicate that zein concentration and the O/W ratio are the primary factors influencing the formation of nanoparticles. It is noteworthy that the study resulted in the production of nanoparticles with an average diameter of approximately 230 nm, a low PDI (indicating uniform PS distribution), and a ZP of around −25 mV, which ensured suspension stability. The results of the PCA and HCA analyses corroborated the presence of different groups of nanoparticles with similar characteristics, thereby reinforcing the importance of adjusting the formulation factors to achieve the desired properties. The composite desirability of 0.9338 corroborates that the response targets were met, thus enabling the determination of the optimal formulation conditions. Stability assessments indicated that refrigeration at 8 °C effectively preserved the nanoparticles’ integrity over 180 days, preventing aggregation and ensuring consistent release profiles. However, a reduction in insulin content was observed, suggesting the need for further optimization to prevent premature release or degradation. Future studies should focus on in vivo evaluations to validate the therapeutic efficacy and safety of these nanoparticles, exploring additional modifications to enhance the EE and stability under physiological conditions. In conclusion, this study presents a comprehensive framework to produce zein-based nanoparticles, emphasizing their potential for controlled drug delivery systems. The optimized formulations provide a promising avenue for enhancing the therapeutic efficacy and stability of encapsulated hydrophilic compounds, particularly in the context of oral administration.

## Supporting Information Available

Table S1, matrix of the 2^4^ factorial design generated using Minitab software; Figure S1, PS normal probability residual plot and histogram of PS residuals; Figure S2, normal probability plot of PDI residuals and histogram of PDI residuals; Figure S3, ZP normal probability residual plot and histogram of ZP residuals; and quantification of insulin by reverse-phase HPLC (PDF)

## Notes

During the preparation of this paper, the author(s) used ChatGPT to improve the readability and language of the manuscript. After using this tool/service, the author(s) have revised and edited the content as necessary and take full responsibility for the content of the published article.
