# Supplementary material for: Optimization of Zein-Casein-Hyaluronic Acid Nanoparticles Obtained by Nanoprecipitation Using Design of Experiments (DoE)
Source: ACS Omega. 2025 Mar 28;10(13):13440–52. doi: 10.1021/acsomega.4c11636 (PMC11983202; doi:10.1021/acsomega.4c11636)
Supplement: Supplementary file 1 — ao4c11636_si_001.pdf [file ao4c11636_si_001.pdf]

Optimization of Zein-Casein-Hyaluronic acid Nanoparticles Obtained by Nanoprecipitation Using Design of Experiments (DoE)

Tatiane Patrícia Babinski[a]; Ariane Krause Padilha Lorenzett[a]; Jeferson Ziebarth[a]; Vanderlei Aparecido de Lima[b]; Rubiana Mara Mainardes [a,c*]

[a]Laboratory of Nanostructured Formulations, Universidade Estadual do Centro-Oeste, Élio Antonio Dalla Vecchia St, 838, 85040-167, Guarapuava, PR, Brazil.

[b]Chemistry Department, Universidade Tecnológica Federal do Paraná, Zip Code 85503-390, Pato Branco, PR, Brazil.

[c]Department of Pharmacy, Universidade Estadual do Centro-Oeste, Élio Antonio Dalla Vecchia St, 838, 85040-167, Guarapuava, PR, Brazil.

*: mainardes@unicentro.br

Table S1. Matrix of the 2^4 factorial design generated using Minitab software.

**Formulation**	**X1**	**X2**	**X3**	**X4**
**F1**	0	0	0	0
**F2**	+1	-1	-1	+1
**F3**	+1	+1	+1	-1
**F4**	+1	+1	+1	+1
**F5**	-1	+1	+1	-1
**F6**	-1	-1	+1	+1
**F7**	+1	-1	+1	-1
**F1**	0	0	0	0
**F1**	0	0	0	0
**F8**	-1	-1	-1	-1
**F9**	-1	+1	-1	+1
**F10**	-1	+1	-1	-1
**F1**	0	0	0	0
**F1**	0	0	0	0
**F11**	-1	-1	+1	-1

**F12**	+1	+1	-1	+1
**F13**	+1	-1	-1	-1
**F14**	+1	+1	-1	-1
**F15**	+1	-1	+1	+1
**F1**	0	0	0	0
**F16**	-1	-1	1	+1
**F17**	-1	+1	1	+1

Figure S1. (a) Particle size normal probability residual plot (b) Histogram of particle size residuals.

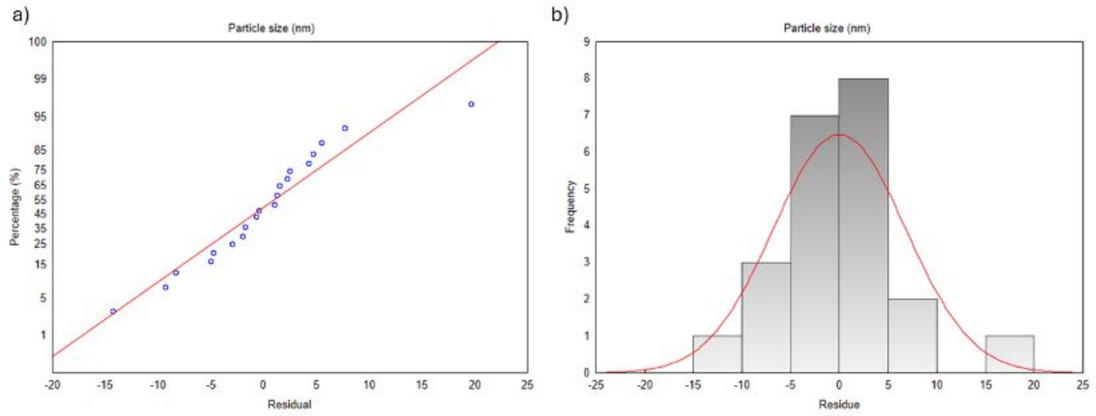

Figure S2. (a) Normal probability plot of PDI residuals (b) Histogram of PDI residuals.

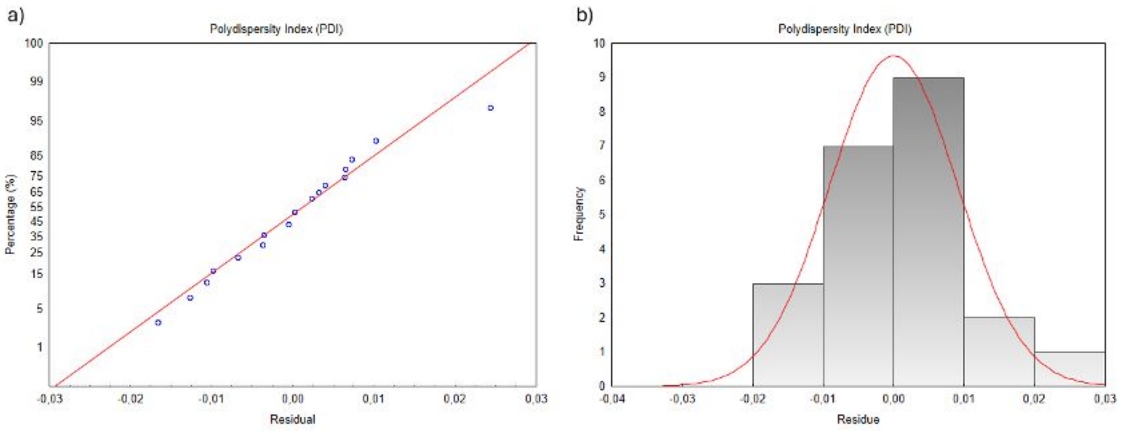

Figure S3. (a) Zeta potential normal probability residual plot. (b) Histogram of zeta potential residuals.

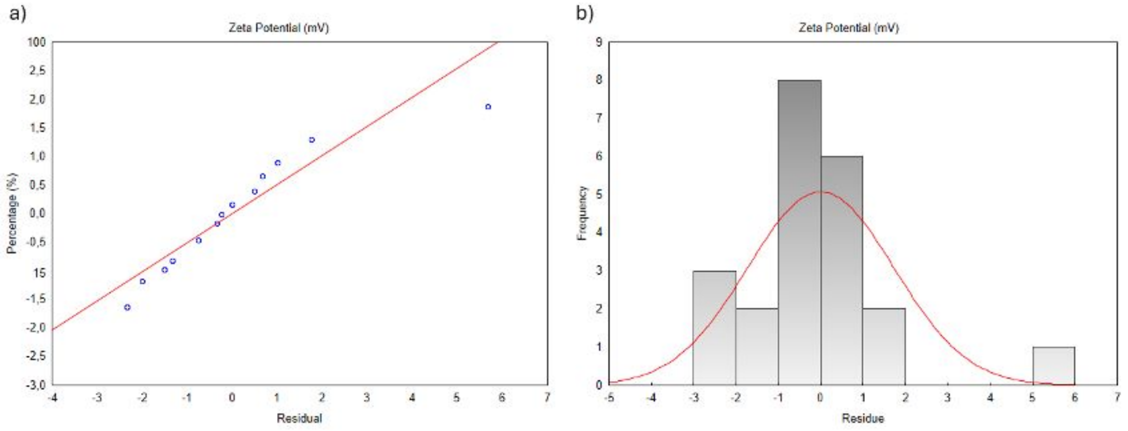

Quantification of Insulin by Reverse Phase High-Performance Liquid Chromatography

Tests for the quantification of Insulin Regular were conducted on a Waters Alliance model liquid chromatograph (e2695) equipped with a Diode Array Detector (DAD), following a methodology developed in accordance with the parameters established [1–3]. The chromatographic conditions used are detailed in Table S2.

Table S2. Chromatographic conditions for quantifying regular insulin in nanoparticles

**Parameters**	**Chromatographic conditions**
Mobile Phase:	Acetonitrile/acidified water * (60:40) (v/v)
Injection volume:	20 µL
Flow:	1mL/min.
Temperature:	30ºC
Detector:	Ultraviolet (uV) λ= 271nm
Chromatographic column:	Atlantis T3
Running time:	6 minutes

*H2O acidified with 0.5% formic acid

The initial chromatographic parameters for the development of the method were established on the basis of information obtained from a literature review [4–8]. To determine the ideal composition of the mobile phase, initial tests were carried out in isocratic mode,

using acetonitrile (ACN) and ultrapure water acidified with 0.5% formic acid (v/v). During these tests, the proportions of solvents were varied in order to find a more regular and symmetrical chromatography peak.

Various ratios were evaluated, including 70:30, 40:60 and 30:70 (v/v). However, the best mobile phase was the 60:40 (v/v) ratio. The retention time for regular insulin was approximately 4.70 minutes, with a mobile phase flow rate set at 1 mL/min. Based on this data, the total run time was set at 6 minutes, as shown in figure S4. For the detection and quantification of regular insulin using DAD, a wavelength of 272 nm was chosen. Based on this selection, all subsequent analyses were carried out at this wavelength.

Figure S4 - Chromatogram obtained at 272 nm of INS standard sample (50 µg/mL) (A); UV-DAD spectrum at retention time of 4.70 minutes (B).

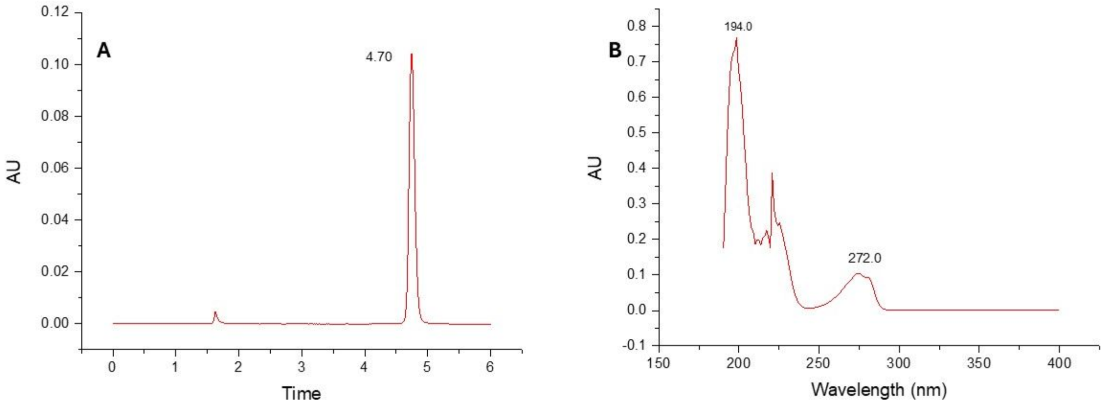

The following parameters were evaluated for validation: linearity, selectivity, limit of detection (LOD), limit of quantification (LQ), precision and accuracy.

- Linearity

The linearity study aims to verify whether the responses obtained are directly proportional to the concentration of the analyte present in the sample, within a predefined range. To this end, an analytical curve was constructed using standard solutions of regular insulin at concentrations of 2, 5, 10, 20, 30, 40 and 50 µg.mL^{-1}, dissolved in acetonitrile as a solvent. The analyses were carried out in triplicate.

The area under the peaks generated was used to calculate the equation of the line and the correlation coefficient (r) of the analytical curve, which are fundamental parameters for assessing its quality. To be considered acceptable, the correlation coefficient must be greater than 0.99.

Linearity is an essential parameter to demonstrate the method's ability to provide results proportional to the concentration of the analyte. The analysis was carried out based on a linear model fit by linear regression, considering a 95% confidence level. The results, which include the equation of the line and the r values, are shown in Table S3.

Table S3 - Analysis of variance (ANOVA) for linear model at 95% confidence level.

**Equation (Linear range): y = 819 + 13514.6 x<br>r: 1.0**			
**Regression**		**Lack of fitting**	
**F observed**	**p-value**	**F calculated**	**p-value**
597489.70	0.000	2.04	0.134

According to the table, ANOVA indicates that the linear model is appropriate for establishing the relationship between chromatographic peak area and concentration. This is evidenced by the F value of the regression (597489.70), which is significantly higher than the tabulated F value for a 95% confidence level (4.35). Furthermore, the model showed no lack of fit ($p>0.05$, with $p=0.134>0.05$). Another important point is that the F value of the calculated regression (2.04) is lower than the tabulated F value (2.99), reinforcing the adequacy of the model. Figure S5 shows the linear regression line obtained from the INS calibration curve, at a 95% confidence level.

Figure S5. INS analytical curve (peak area versus INS concentration). Equation of the line: y = 819 + 13514.6 x (r =1.0).

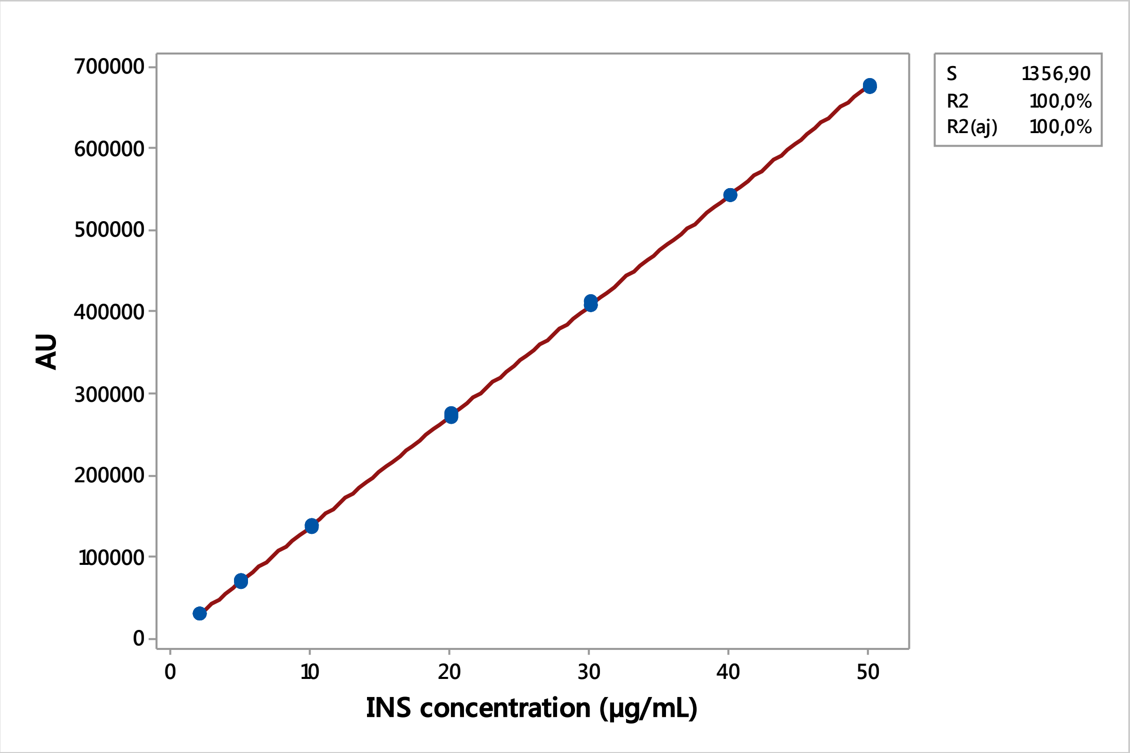

The r value obtained for INS was 1.0, demonstrating the quality and reliability of the analytical curve. The closer the r value is to 1, the lower the dispersion of the experimental points and the more reliable the determination of analyte concentrations will be based on the established method[1,9]. Thus, the linearity of the curves guarantees the quality of the method for analyzing INS, ensuring confidence in the use of the straight line equation obtained.

- Selectivity

Selectivity is an essential parameter for identifying the analyte of interest in the midst of other components present in the formulation, such as impurities, matrix constituents or degradation products. To assess the potential interference of the compounds used in the formulation of NPs in the determination of regular insulin, tests were carried out with the supernatant obtained from the preparation of NPs without insulin (empty NPs).

The analysis was carried out by comparing the chromatograms of the supernatant of the empty NPs with those of the NPs loaded with regular insulin. This comparison included the evaluation of retention times and chromatographic peaks, allowing a careful analysis of the method's selectivity. In addition, a chromatogram of a standard solution containing 50 µg.mL^{-1} of regular insulin was obtained. The chromatogram of the standard

was compared to that of the supernatant of the loaded NPs, analyzing both the retention times and the UV-DAD spectra of the standard and the sample.

This procedure guaranteed the selectivity of the analytical method, ensuring reliability in the determination of regular insulin even in the presence of potentially interfering substances. Components such as Zn, HA and CS, which make up the NPs, can generate unwanted peaks close to the insulin retention time, making analysis difficult.
To identify possible interfering peaks, the supernatants of the uncharged and charged NPs were analyzed. After centrifugation, the supernatants were diluted in acetonitrile and subjected to high-performance liquid chromatography (HPLC) under the previously established conditions.

Figure S6 shows the chromatogram obtained for the selectivity analysis, comparing the peak of the INS standard solution with the peaks obtained from both the charged and uncharged nanoparticles. This provides a clear view of the differences and particularities of these samples.

Figure S6. Chromatogram of supernatant samples of empty NPs, NPs loaded with INS, and INS standard (50 µg/mL).

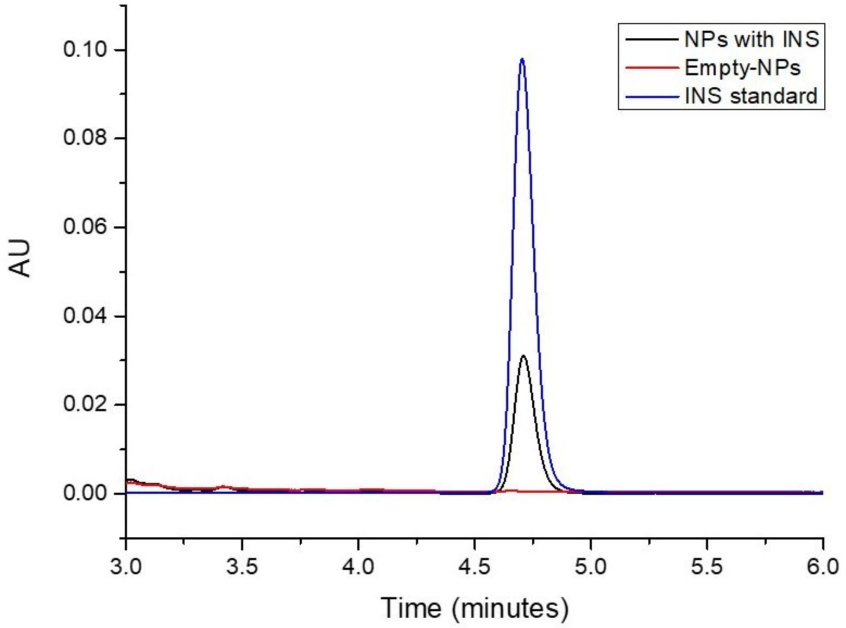

As can be seen, in both chromatograms it is possible to observe that the charged nanoparticle showed a characteristic peak of the drug INS at a retention time of 4.70, which is in line with the profile obtained with the standard. Meanwhile, the empty NPs

showed no interfering peaks at the same retention time, indicating that there was no interference in the quantitative determination of INS from the formulation components.

Figure S7 shows the UV-DAD spectrum obtained at 272 nm of the INS standard solution and the supernatant of the loaded NPs.

Figure S7 - INS standard UV-DAD spectrum (A); INS loaded NPs UV-DAD spectrum (B).

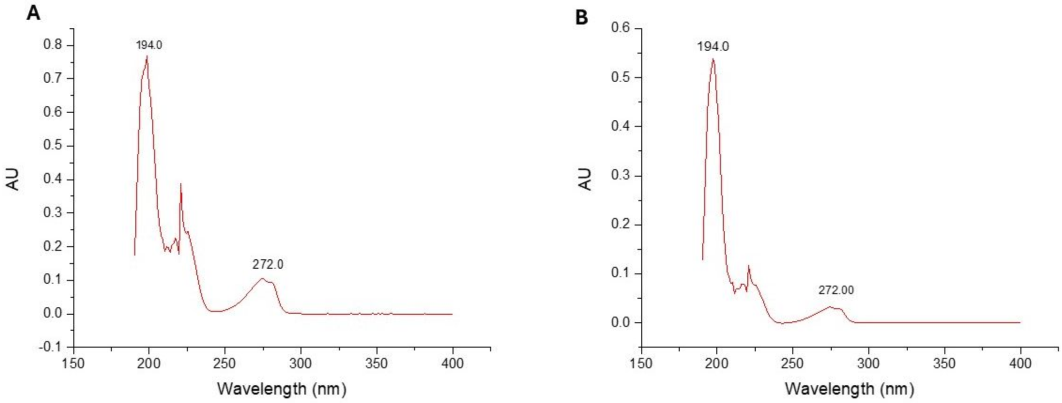

A comparison of the two spectra shows that the method can identify insulin even in the supernatant of the loaded NPs. The similarities in the peaks indicate that the method is selective for INS, despite the changes in intensity, which reflect variations in the amount of INS detectable in the medium. This suggests that the analytical method can be reliable for distinguishing encapsulated insulin from other components present in NPs. Based on the results obtained, it is possible to state that the proposed method showed adequate selectivity for the analysis of INS in the NPS supernatants developed.

- Limits of Detection (LD) and Quantification (LQ)

The definition of the Limit of Detection (LOD) refers to the lowest concentration of the sample being analyzed that can be detected, although not necessarily quantified. On the other hand, the Limit of Quantification (LQ) is the lowest concentration of the sample that can be measured with acceptable precision and accuracy, according to the experimental conditions established[2].

Equations 1 and 2 were used to determine the LOD and LQ values, based on the data provided by the analytical curve constructed to assess linearity.

LD= σ/b x 3.3 (Eq.1) LQ= σ/b x 10 (Eq.2)

Where:

σ= Average standard deviation of the intercept with the y-axis;

b= Slope of the analytical curve

The limits of detection (LD) and quantification (LQ) were determined from the equation of the straight line, using equations 1 and 2 in section 3.3, resulting in values of 0.11 µg/mL and 0.36 µg/mL, respectively. These results demonstrate the high sensitivity and reliability of the method, allowing precise detection and quantification of the analyte at low concentrations. Furthermore, the values obtained are in agreement with those reported in the literature. Moslemi et al. (2003) described an LD of 0.25 µg/mL and a LQ of 0.75 µg/mL, using a similar chromatographic method[10]. These findings reinforce the suitability and robustness of the method proposed in this study, demonstrating its applicability for analysis at low concentrations of the analyte.

- Precision

Accuracy was assessed using repeatability (intra-run) and intermediate accuracy (inter-run) tests, using analysis of known concentrations (5, 20 and 40 µg/mL). These concentrations were injected in triplicate over three days. The results obtained were used to calculate the relative standard deviation (RSD) by applying equation 3.

RSD %= SD/CMD x 100 (Eq.3)

Where:

SD= Standard deviation;

CMD= Average concentration determined.

Precision is expressed as RSD% (Relative Standard Deviation), which represents the measure of the method's relative error. To check the method's precision, repeatability (intra-run) analyses were carried out on the same day and intermediate precision (inter-run) on three consecutive days. The results of these analyses are shown in Table S4.

Table S4 - Intra and inter race accuracy results

**INTRA RACE PRECISION**		
**Theoretical concentration (µg.mL-1)**	**Mean measured concentration (µg.mL-1) ± SD**	**RSD (%)**
5	5.09 ± 0.1	3.19
20	20.57 ± 0.5	2.64

40		41.17 ± 1.2		2.87
**INTER-RACE PRECISION**				
**Theoretical concentration** (**μg.mL^{-1}**)	**Day 1** (**μg.mL^{-1}**) **± DP**	**Day 2** (**μg.mL^{-1}**) **± DP**	**Day 3** (**μg.mL^{-1}**) **± DP**	**RSD (%)**
5	5.3 ± 0.2	5.3 ± 0.2	5.2 ± 0.1	1.13
20	21.1 ± 0.1	21.2 ± 0.2	20.6 ± 0.3	1.66
40	42.3 ± 0.1	42.1 ± 0.2	41.3 ± 0.1	1.13

It can be seen that all DPR values are within the limit required by literature, as recommended by ANVISA (2003) and ICH (2005), which establish a maximum limit of 5% [1,2] . The highest RSD value found was 3.19%, corresponding to the intra-run analysis of the INS standard sample at a concentration of 5 μg/mL, which is still considered an appropriate value, being below the 5% limit. Therefore, it can be concluded that the proposed HPLC-DAD method has adequate precision for the analysis of INS in the supernatants of the Nps developed.

- Accuracy

Accuracy is defined as the degree of agreement between the results obtained in a test and a reference value considered to be true. To assess this, tests were carried out using the same concentrations as in the precision analysis (5, 20 and 40 μg/mL), with three replicates for each concentration. The results were expressed in terms of percentage recovery, calculated using the formula in equation 4:

% Recovery= EMC/ TC x 100 (Eq.4)

Where:

EMC= Experimental mean concentration;

TC= Theoretical concentration.

The accuracy of the chromatographic method was assessed based on the recovery of the insulin standard in a solution containing all the constituents of the system. Recovery tests were conducted by adding the insulin standard at three different concentrations (5, 20 and 40 μg/mL) to the supernatant of the NPs. The percentage recovery was determined using the equation mentioned above, and the results are shown in Table S5.

Table S5 - Standard addition recovery values.

**Theoretical concentration (µg.mL-1)**	**Recovery percentage (%) ± SD**
5	103.83 ± 1.08
20	102.85 ± 0.09
40	103.30 ± 0.04

As shown in the table above, the values obtained for the percentage of recovery in all the concentrations analyzed are in line with the regulations. These values are accepted by ANVISA (2003), which establish acceptance ranges of 95 to 105% and 80 to 110%, respectively[1]. Therefore, we can state that the proposed method shows accuracy within the acceptance parameters.

References

(1) Ministério Da Saúde Agência Nacional de Vigilância Sanitária RESOLUÇÃO DA DIRETORIA COLEGIADA-RDC No 166, DE 24 DE JULHO DE 2017.

(2) International conference on harmonisation of technical requirements for registration of pharmaceuticals for human use ich harmonised tripartite guideline validation of analytical procedures: text and methodology q2(R1).

(3) *DOQ-CGCRE-008 Revisão 09 — Centro de Desenvolvimento da Tecnologia Nuclear - CDTN.* https://www.gov.br/cdtn/pt-br/assuntos/documentos-cgcre-abnt-nbr-iso-iec-17025/doq-cgcre-008/view (accessed 2025-02-25).

(4) Najjar, A.; Alawi, M.; AbuHeshmeh, N.; Sallam, A. A Rapid, Isocratic HPLC Method for Determination of Insulin and Its Degradation Product. *Advances in Pharmaceutics* 2014, *2014*, 1–6. https://doi.org/10.1155/2014/749823.

(5) Wahl, O.; Jorajuria, S. Development and Validation of a New UHPLC Method for Related Proteins in Insulin and Insulin Analogues as an Alternative to the European Pharmacopoeia RP-HPLC Method. *J Pharm Biomed Anal* 2019, *166*, 71–82. https://doi.org/10.1016/j.jpba.2018.12.034.

(6) Wahl, O.; Jorajuria, S. Development and Validation of a New UHPLC Method for Related Proteins in Insulin and Insulin Analogues as an Alternative to the European

Pharmacopoeia RP-HPLC Method. *J Pharm Biomed Anal* 2019, *166*, 71–82. https://doi.org/10.1016/j.jpba.2018.12.034.

(7) Kristl, A.; Podgornik, A.; Pompe, M. Simultaneous Separation of Insulin and Six Therapeutic Analogues on a Mixed Mode Column: HPLC-UV Method Development and Application. *J Chromatogr B Analyt Technol Biomed Life Sci* 2021, *1171*. https://doi.org/10.1016/j.jchromb.2021.122557.

(8) Shen, Y.; Prinyawiwatkul, W.; Xu, Z. Insulin: A Review of Analytical Methods. *Analyst*. Royal Society of Chemistry July 21, 2019, pp 4139–4148. https://doi.org/10.1039/c9an00112c.

(9) Ribani, M.; Grespan Bottoli, C. B.; Collins, C. H.; Fontes Jardim, I. C. S.; Costa Melo, L. F. Validação Em Métodos Cromatográficos e Eletroforéticos. *Quim Nova* 2004, *27* (5), 771–780. https://doi.org/10.1590/S0100-40422004000500017.

(10) Moslemi, P.; Najafabadi, A. R.; Tajerzadeh, H. A Rapid and Sensitive Method for Simultaneous Determination of Insulin and A21-Desamido Insulin by High-Performance Liquid Chromatography. *J Pharm Biomed Anal* 2003, *33 1* (1), 45–51. https://doi.org/10.1016/S0731-7085(03)00336-4.
